# Water Dispersible Few-Layer Graphene Stabilized by a Novel Pyrene Derivative at Micromolar Concentration

**DOI:** 10.3390/nano8090675

**Published:** 2018-08-30

**Authors:** Eunice Cunha, Maria Fernanda Proença, Maria Goreti Pereira, Maria José Fernandes, Robert J. Young, Karol Strutyński, Manuel Melle-Franco, Mariam Gonzalez-Debs, Paulo E. Lopes, Maria da Conceição Paiva

**Affiliations:** 1 Institute for Polymers and Composites/i3N, University of Minho, Campus of Azurém, 4800-058 Guimarães, Portugal; eunice.cunha-2@manchester.ac.uk (E.C.); mjfernandes@iol.pt (M.J.F.); pelopes@dep.uminho.pt (P.E.L.); 2 National Graphene Institute and School of Materials, University of Manchester, Manchester M13 9PL, UK; robert.young@manchester.ac.uk; 3 Centre of Chemistry, University of Minho, Campus of Gualtar, 4710-057 Braga, Portugal; fproenca@quimica.uminho.pt (M.F.P.); goreti.pereira@ufpe.br (M.G.P.); 4 CICECO-Aveiro Institute of Materials, Department of Chemistry, University of Aveiro, 3810-193 Aveiro, Portugal; skarol@ua.pt (K.S.); manuelmelle@ua.pt (M.M.-F.); 5 Microfabrication and Exploratory Nanotechnology Department, International Iberian Nanotechnology Laboratory (INL), 4715-330 Braga, Portugal; mariam.debs@inl.int

**Keywords:** few layer graphene, pyrene derivative, aqueous suspension, molecular modelling

## Abstract

The search for graphene or few-layer graphene production methods that are simple, allow mass production, and yield good quality material continues to provoke intense investigation. The present work contributes to this investigation through the study of the aqueous exfoliation of four types of graphene sources, which are namely graphite and graphite nanoflakes with different morphologies and geographical origins. The exfoliation was achieved in an aqueous solution of a soluble pyrene derivative that was synthesized to achieve maximum interaction with the graphene surface at low concentration (5 × 10^−5^ M). The yield of bilayer and few-layer graphene obtained was quantified by Raman spectroscopic analysis, and the adsorption of the pyrene derivative on the graphene surface was studied by thermogravimetric analysis and X-ray diffraction. The whole procedure was rationalized with the help of molecular modeling.

## 1. Introduction

Graphene has emerged as an exciting material that has been intensively studied throughout the last decade, revealing potential applications in various fields [1,2,3]. However, the extrapolation of its outstanding properties observed on the lab-scale into large-scale industrial applications is limited by the lack of effective methods for the large scale production of good quality graphene. The liquid phase exfoliation (LPE) of graphite is a potentially viable cost-effective process that can be upscaled to the mass production of graphene and few layer graphene (FLG). Typically, the LPE processes occur in organic solvents, although their use in large volumes has negative environmental consequences. Water is a “green” solvent, and its use in this process is a step to overcome this problem. However, it has a high surface tension (72 mJ m^−2^), which limits its interaction with graphite and graphene, and the ability to form stable suspensions. The hydrophobic nature of graphene leads to its re-stacking and agglomeration in aqueous dispersion. In fact, solvents with surface tensions of near 40 mJ m^−2^ [4] are reported to be ideal for the exfoliation of graphite into graphene [5].

The performance of water for the LPE of graphite can be greatly improved with the aid of amphiphilic molecules that strongly interact with water and with graphene simultaneously. These molecules help the dispersion of graphite flakes and graphene, preventing their agglomeration. Polycyclic aromatic hydrocarbons such as pyrene derivatives have been studied for the exfoliation and stabilization of graphene and FLG in water [6,7]. The adsorption of pyrene derivative molecules onto the graphene surface occurs via π-π interactions between the planar π-conjugated surfaces, while hydrophilic functional groups attached to the pyrene moiety allow their stabilization in aqueous media. Compounds such as pyrenebutyrate (PB^−^) and pyrene sulfonic acid salt, as well as a pyrene-terminated poly(2-N,N′-(dimethyl amino ethyl acrylate) (PDMAEA) and poly(acrylic acid) (PAA) have been investigated for use in solar cells, as well as electrochemical and composites applications [8,9,10]. In these studies, reduced graphene oxide (r-GO) was initially prepared from graphene oxide (GO) and then stabilized in water. 

Other approaches, based on the direct exfoliation of graphite in water, have been reported. A pyrene-functionalized amphiphilic block copolymer, poly(pyrenemethyl acrylate)-*b*-poly((polyethylene glycol) acrylate) (polyPA-*b*-polyPEG-A) and a pyrene-terminated polyethylene glycol (pyrene-PEG) were used by Liu et al. [11] and Zheng et al. [12], respectively, to exfoliate graphite in water and produce nanocomposites with enhanced mechanical and thermal properties.

Biocompatible polymers have been functionalized with pyrene moieties to prepare aqueous graphene suspensions for biomedical applications [13,14,15]. Pyrene derivatives were used to directly exfoliate graphite in water without bonding with polymer chains. The process allows the formation of stable water suspensions of graphene and few-layer graphene, which has been reported for applications in areas such polymer composites, sensors, and energy storage [16,17,18]. Typically, aminopyrene, aminomethyl pyrene, pyrene carboxylic acid, pyrene butyric acid, and pyrene sulfonic acid derivatives have been used in the graphite exfoliation process at concentrations between 0.1–4.0 mg/mL (0.1 mM to 10 mM). [19,20,21,22,23] These concentrations are normally above the critical aggregation concentration for pyrene derivatives, causing them to self-assemble in solution [24] (e.g., the critical aggregation concentration for 1-pyrenebutyric acid is 0.1 mM) [25] and requiring long sonication times that are reported to severely reduce the flake size of graphene as well as increase structural defects [6,26,27]. The more common pyrene derivatives reported in the literature for the study of the exfoliation and stabilization of graphene in water are commercially available products. The cost of these compounds may be a limitation for their large-scale application.

In this paper, the synthesis of a pyrene derivative through a simple and low-cost functionalization methodology is presented. This synthesis presents a high yield of the modified pyrene that may be scaled up for large-scale production. The synthetized pyrene derivative is soluble in water, and it was tested for the exfoliation and stabilization of three different types of graphite nanoplatelets (GnP) [28], which were obtained by different processes, and natural graphite in aqueous media, at low pyrene concentrations (0.05 mM). This procedure allowed the stabilization of FLG (<10 layers) in water with interesting yield, produced from all of the GnP as well as from graphite. The exfoliation products were characterized by Raman and UV-Vis spectroscopies, and the concentration of few-layer graphene obtained in suspension was measured. The uptake of pyrene derivative by the graphene in suspension was evaluated by thermogravimetric analysis, and the results were analyzed with the aid of molecular modeling. The exfoliation of the pristine graphite material was analyzed by X-ray diffraction. The very low concentration of pyrene derivatives used in this work (which to our knowledge are the lowest reported in the literature) to exfoliate graphite in water opens up good potential for an efficient process to obtain FLG.

## 2. Materials and Methods

### 2.1. Materials

Two grades of GnP were purchased from XG Sciences (Lansing, MI, USA), grade C (GnPC), and grade H5 (GnPH5). GnPC has, according to the manufacturer, a size distribution ranging from very small (100 nm) to relatively large flakes (1–2 µm), an average thickness of approximately 2 nm, and a typical average surface area of 750 m^2^/g, while GnPH5 has a nominal equivalent diameter of 5 µm, thickness of 15 nm, and an average surface area of 60–80 m^2^/g. GnP Micrograf, provided by Nacional de Grafite (grade Micrograf HC11, São Paulo, Brasil), is a micronized graphite with an equivalent diameter of about 10 µm, and Graphexel is a natural crystalline graphite with a large equivalent diameter of approximately 180 µm that was provided by Graphexel Ltd. (graphexel grade 2369, Essex, UK). Copper (II) nitrate trihydrate (Cu(NO_3_)_2_·3H_2_O), anhydrous copper (II) sulphate (CuSO_4_), and potassium hydroxide pellets (KOH) were purchased from Sigma Aldrich (Sintra, Portugal). Maleic anhydride 99% pellets and pyrene 98% were purchased from Acros Organics (Geel, Belgium). 4-nitrobenzaldehyde was obtained from Merck (Sintra, Portugal). Acetic anhydride (Ac_2_O) and sodium borohydride (NaBH_4_) were purchased from VWR chemicals (Radnor, PA, USA). Ethyl acetate (EtOAc), petroleum ether 40–60 °C, ethanol absolute (EtOH) and acetonitrile (ACN) were purchased from Fisher Scientific (Hillsboro, Oregon). Anhydrous magnesium sulphate (MgSO_4_), dicholoromethane (CH_2_Cl_2_), and diethyl ether were obtained from Panreac (Barcelona, Spain). Deuterated dimethyl sulfoxide (DMSO-d_6_, 99.80%) was purchased from Euriso-top (Saint Aubin, France).

**Figure 1:**
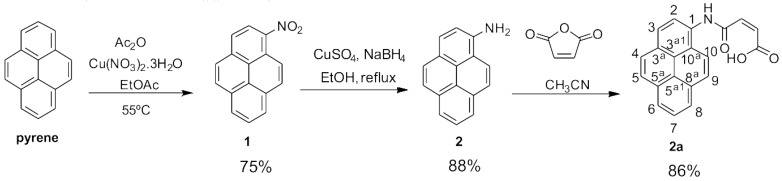
Schematic representation of the pyrene derivative synthesis.

The water-soluble pyrene derivative was prepared from pyrene in three steps (Figure 1): first, by nitration (compound 1) using copper nitrate [29], followed by its reduction with sodium borohydride (compound 2), and finally, the 1-aminopyrene 2 was directly combined with maleic anhydride, generating a carboxylic acid group three carbon atoms away from the pyrene moiety (compound 3). This pyrene derivative was fully characterized by Fourier transform infrared spectroscopy (FTIR) spectroscopy and by ^1^H and ^13^C nuclear magnetic resonance (NMR) (including the bidimensional technique of Heteronuclear Single Quantum Correlation (HSQC)). The detailed information about the synthesis of the pyrene derivatives and its spectral characterization (Figures S2 and S4) are given in the supporting information.

### 2.2. Preparation and Characterization of the Exfoliated Graphite Suspension 

The pyrene derivative was dissolved in distilled water at 0.05-mM concentration, and the pH was adjusted to seven by the addition of potassium hydroxide (KOH). Graphite suspensions were prepared by mixing approximately 5.0 mg of graphite in 10 mL of the pyrene derivative solution using an Ultrasonic processor UP100H from Hielscher (Teltow, Germany) equipped with a sonotrode MS7D. Ultrasound energy was applied to the suspensions for 1 h at maximum power. The suspensions were centrifuged (8000 rpm, 1 h) to remove larger aggregates, and the supernatant was collected. These stable suspensions were analyzed by UV-visible spectroscopy on a Shimadzu UV-2401PC, using quartz cells with a 10-mm pathlength. The concentration was determined using the Beer–Lambert Law, and the extinction coefficient for these materials was calculated using a procedure reported elsewhere [5].

The suspensions were sprayed on a glass slide using a XL2000 Airbrush (Graphics Direct, York, UK) with a 0.6-mm nozzle and analyzed by Raman spectroscopy. Raman spectra were obtained on a Horiba LabRAM HR Evolution confocal microscope (Horiba Scientific, Longjumeau, France) using a laser excitation of 532 nm (2.33 eV). A 100× objective lens was used to focus the laser onto the sample. For each sample, 60 spectra were collected randomly over a sample area of 50 × 20 mm, and analyzed using the LabSpec 6 software (also from Horiba Scientific). Lorentzian functions were used to fit the characteristic peaks of the spectra.

Scanning transmission electron microscopy (STEM) samples were prepared by the deposition of liquid suspensions of exfoliated graphite samples on 400 mesh carbon coated copper grids (CF400-Cu, Electron Microscopy Sciences, Hatfield, PA, USA) and dried using a hot plate. The samples were analyzed using a NanoSEM FEI Nova 200 microscope (ThermoFisher Scientific, Hillsboro, Oregon). The dried samples, which were obtained by the solvent evaporation of the liquid suspensions of exfoliated graphite, as well as the graphite starting material, were analyzed by scanning electron microscopy (SEM) using the same equipment. The thermogravimetric analysis (TGA) of these powder samples was performed on a Modulated TGA Q500 from TA Instruments (Newcastle, DE, USA), heating the samples at 10 °C/min under a constant flow of N_2_.

X-rays diffraction (XRD) measurements were performed on a PANalytical X´Pert PRO XRD System (Malvern, UK) using Cu–Kα radiation with the X-ray tube operated at 45 kV and 40 mA. The XRD spectra were obtained by continuously scanning 2theta/omega axes in steps of 0.0098 2theta°, using a scanning line detector with Soller slits and a Ni filter. The X-ray beam was incident over the sample in line focus type using a 1° divergence slit and also Soller slits. The XRD samples were obtained from solvent drying from the bulk solution, powder samples, and by spraying the solution over a heated silica wafer.

## 3. Results and Discussion

In the present work, an amphiphilic pyrene derivative was synthesized for the purpose of graphite exfoliation, combining the pyrene moiety for an effective π-π interaction with the graphene layers and a hydrophilic carboxylic group for affinity with water (structure 3 in Figure 1).

### 3.1. Synthesis of the Pyrene Derivative

The structure of compound 3 was confirmed by the presence of the amide and the carboxylic acid protons at δ10.84 and 13.26 ppm, respectively, in the ^1^H NMR spectrum. The cis configuration of the exocyclic alkene was supported by the coupling constant between both protons of this functional group (J = 12.1 Hz), which is typical for this isomer [30]. The two carbonyl groups were visible in the ^13^C NMR spectrum at δ 164.46 ppm (for the amide) and δ 166.84 ppm (for the carboxylic acid). The FT-IR spectra are also presented as supporting information (Figures S1, S3 and S5). Compound 1 presents the two characteristic bands of the nitro groups at 1506 cm^−1^ and 1331 cm^−1^, corresponding to the –NO_2_ asymmetric and symmetric stretching vibrations. After reduction to 2, the characteristic symmetric and asymmetric stretching vibrations of the primary amino group can be seen at 3445 cm^−1^ and 3379 cm^−1^. The reaction of product 2 with maleic anhydride leads to the final product 3 showing the characteristic intense bands for the stretching vibration of the carbonyl groups at 1711 cm^−1^.

### 3.2. Exfoliation of Graphite

**Figure 2:**
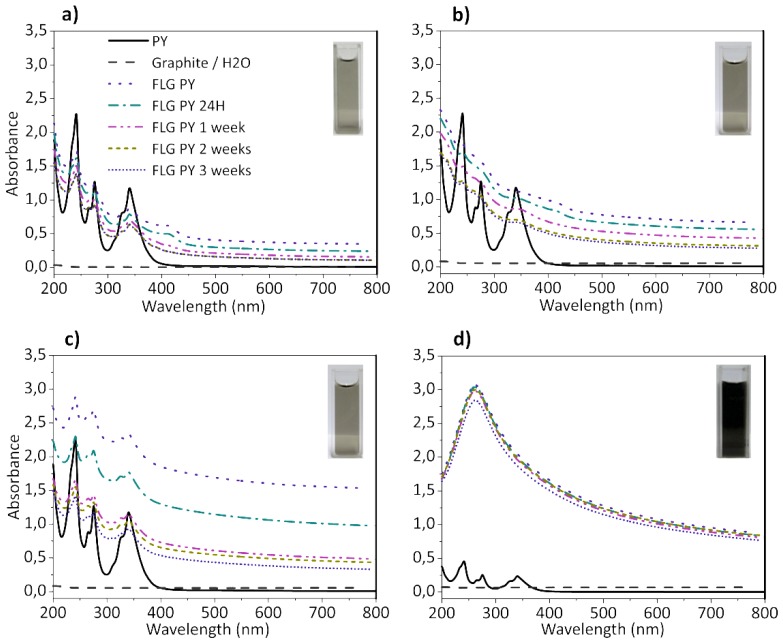
UV-visible spectra of the stable dispersions of exfoliated graphite, as prepared, and of the pyrene derivative solution for: (**a**) Graphexel; (**b**) Micrograf; and (**c**) grade 5 graphite nanoplatelets (GnPH5). (**d**) UV-visible spectra of the 10× diluted grade C graphite nanoplatelets (GnPC) dispersions and the pyrene solution.

The pyrene derivative 3 (PY) was used to exfoliate the graphite nanoplatelets GnPH5, GnPC, Micrograf and the natural graphite Graphexel in water, and stabilize the FLG formed. The same exfoliation/centrifugation procedure was applied to the different graphite grades using distilled water without PY (blank tests: graphite/H_2_O). Figure 2 shows the UV-visible spectra of these dispersions and their comparison with the initial PY solution.

The initial PY solution shows the characteristic peaks between 200–600 nm [20], while the baseline absorption of the FLG dispersions (FLG PY) extends monotonically over the whole wavelength range, which is typical of graphene dispersions [17,31]. Furthermore, the stability of the FLG PY dispersions was evaluated, showing them to be reasonably stable with time, even after three weeks. For the GnPH5 FLG PY, Micrograf FLG PY, and Graphexel FLG PY dispersions, the original peaks of the PY in solution still remain visible, although broader and with lower intensity. A similar observation was reported for single-walled carbon nanotubes dispersed in water/modified pyrene solutions [32]. It is indicative of the π-π interactions between the functionalized aromatic molecules and the exfoliated graphite [20].

**Figure 3:**
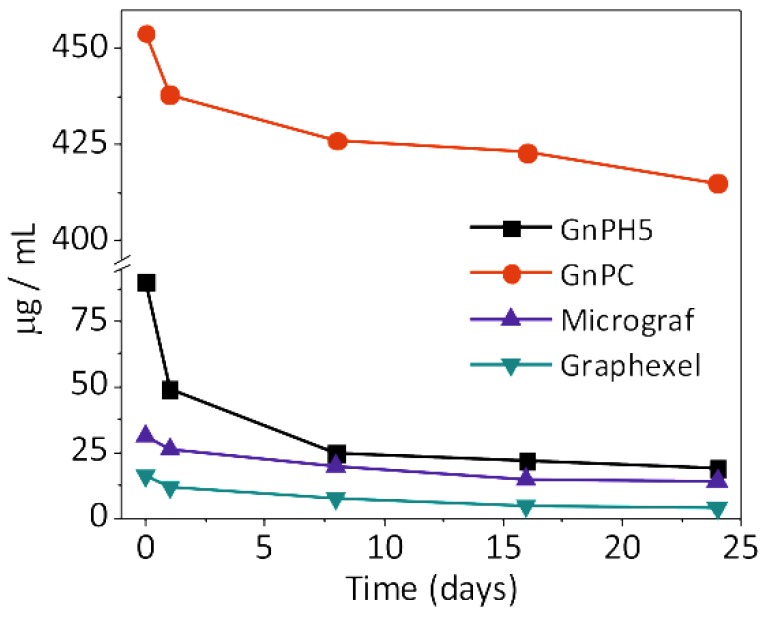
Concentration of few-layer graphene GnPH5, GnPC, Micrograf, and Graphexel in water as a function of time.

The suspensions obtained after centrifugation of the GnPC PY were highly concentrated, showing saturation of the PY absorption peaks. The spectra of the GnPC FLG PY presented in the Figure 2 were obtained for the diluted suspensions (10× dilution), producing an absorption peak with a shape similar to those reported for graphene and graphene oxide in solution [20,33,34]. The absorption observed for the blank tests (Graphite/H_2_O, dashed lines in Figure 2) showed a very low intensity over the whole wavelength range, indicating that the pyrene derivatives play an important role in the stabilization of the exfoliated graphite in water. The extinction coefficient of the exfoliated graphite in water was measured from UV-visible spectra at 660 nm, yielding the value of 2200 ± 100 Lg^−1^ m^−1^. These results are in line with the values reported in the literature [5,20]. Based on the extinction coefficient values obtained, the concentration of exfoliated graphite in suspension was measured over time, and its evolution is represented in Figure 3.

The yield of FLG in suspension was calculated relative to the initial concentration of graphite (approximately 500 µg/mL), and the concentration of FLG was determined by UV-visible spectroscopy. The results are presented in Table 1. The FLG suspension obtained from GnPC presented the highest yield of nanoparticles, which was possibly due to the smaller flake size as well as smaller thickness (~2 nm) compared to the other graphite materials, which facilitated the exfoliation and stabilization in water. Furthermore, according to the producer, GnPC has an atomic concentration of oxygen of approximately 7%, which may also enhance its dispersibility in water. However, in the absence of PY, GnPC is not stable in aqueous suspension (blank test, Figure 2d). The yield of FLG PY in aqueous suspension is higher for GnPH5 and Micrograf relative to Graphexel. GnPH5 and Micrograf are expanded graphite and micronized graphite grades, respectively, with a similar flake geometry, while Graphexel is a natural graphite with larger equivalent diameter and flake thickness, which may limit the yield of exfoliated material in suspension. It is known that flake dimensions and sonication time influence the yield of graphene in suspension [6,27]; typically, larger and thicker flakes lead to lower exfoliation yield, while for a given graphite geometry, higher sonication times induce higher levels of graphite exfoliation. Table 1 summarizes the exfoliation yield achieved in aqueous suspension reported by several authors using pyrene derivatives, as well as the present results (after eight days of stabilization).

### 3.3. Characterization of the Exfoliated Materials

**Figure 4:**
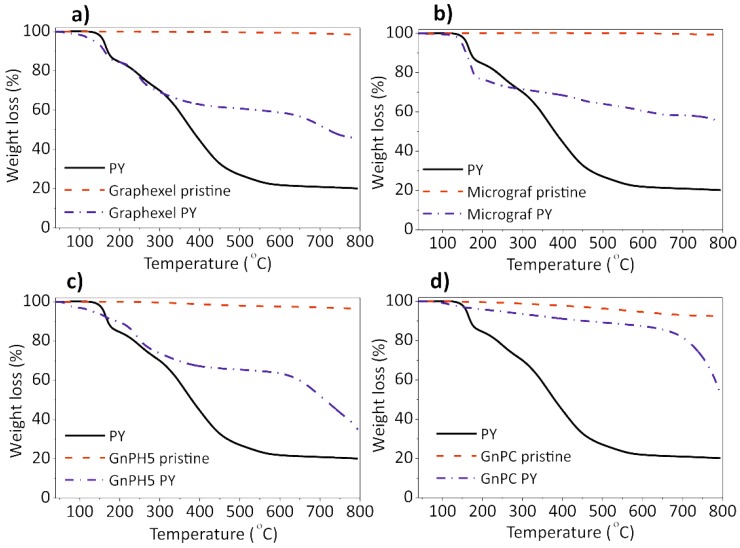
Thermogravimetric analysis (TGA) curves of the graphite starting material and exfoliated graphite: (**a**) Graphexel; (**b**) Micrograf; (**c**) GnPH5; and (**d**) GnPC.

Figure 4 shows the thermogravimetric analysis for the four graphite materials studied, as-received and after PY exfoliation and solvent evaporation, as well as for the PY alone. The PY thermogram is characterized by a first weight loss step near 150 °C, followed by progressive weight loss until a steady residue of 24 ± 2 wt% is reached above 600 °C. Considering the stability of the pyrene molecule up to 400 °C, the initial weight loss at 150 °C may be assigned to a molecular rearrangement in the functional group bonded to the pyrene moiety. The weight loss measured at that temperature is indeed consistent with the loss of one water molecule, which may have resulted from dehydration of the carboxylic acid in PY to yield the corresponding anhydride. The residue remaining at 600 °C may result from a combination of the degradation products of PY at high temperature and inert atmosphere, forming heavier molecules that remain stable at 600 °C. This is consistent with the literature results showing that pyrene derivatives with a carboxylic moiety tend to self-assemble to produce stable structures [24].

The TGA curves of the graphite starting materials Graphexel, Micrograf, GnPH5, and GnPC show thermally stable materials with a weight loss at 600 °C of 0.6 wt%, 0.1 wt%, 2.4 wt% and 5.4 wt%, respectively. GnPC presents a larger weight loss at 600 °C, which may be due to its smaller flake size and decomposition of the oxygen-containing groups. The weight loss of the corresponding FLGs, also at 600 °C, are 39 ± 2 wt%, 35 ± 2 wt%, 34 ± 1 wt%, and 12 ± 2 wt% respectively, and these large values are due to the decomposition of the PY molecules adsorbed on the FLG surface.

**Figure 5:**
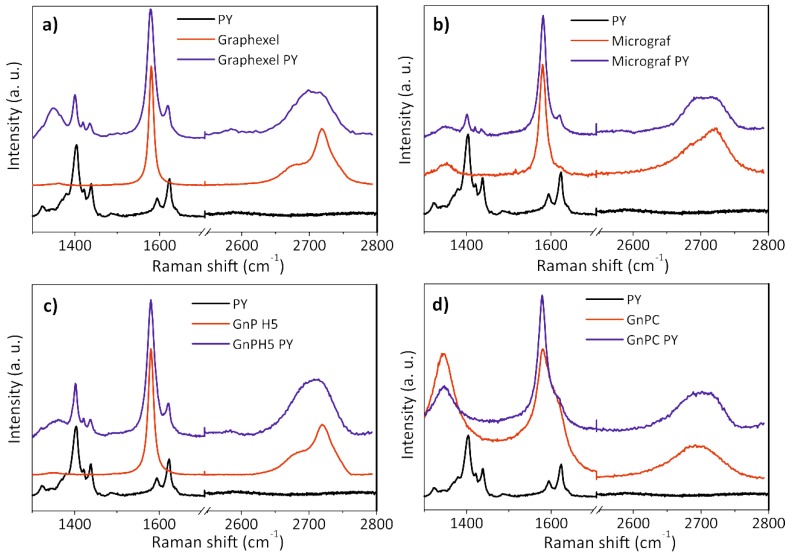
Raman spectra of the pyrene derivative, graphite starting material, and exfoliated graphite: (**a**) Graphexel; (**b**) Micrograf; (**c**) GnPH5; and (**d**) GnPC.

Raman spectroscopy is an important tool for the characterization of graphene and carbon-based materials. The Raman spectrum of single-layer graphene presents three main characteristic bands, associated with the G, D, and 2D (or G′) modes. The G mode, which is observed near 1580 cm^−1^, is present in all of the materials with conjugated C–C double bonds and reflects the in-plane bond stretching motion of pairs of C sp^2^ atoms [35]. Due to the strong C–C bonding in graphene, the G band is observed at a relatively high Raman frequency compared to other materials. The frequency of the G band is invariant relative to the laser excitation energy (E_laser_), but is sensitive to temperature, strain, and doping of the graphene sample. The D mode, which is observed near 1350 cm^−1^, is a breathing mode of A_1g_ symmetry that is forbidden in perfect graphene, becoming active in the presence of disorder [35,36]. Its presence indicates the existence of a hexagonal sp^2^ carbon network disturbed by chemical bonding such as that observed adjacent to a graphene edge or a defect. The D band is highly dispersive as a function of the E_laser_. The two-dimensional (2D) band is a second-order mode that is sometimes referred as an overtone of the D band, although it is not related to graphene defects, and is always strong in graphene, even when the D band is absent. The shape and position of the 2D band varies with the number of graphene layers [37]. For single-layer graphene, it has twice the intensity of the G band, while the bilayer and higher number of layers forms display a G band with higher intensity compared to the 2D band. The 2D band of single-layer graphene may be fitted with a single Lorentzian function with a full width at half maximum (FWHM) near 24 cm^−1^, while bilayer graphene requires four characteristic Lorentzian functions, each with a FWHM of ~24 cm^−1^. As the number of layers increases, the 2D band shifts to a higher wave number and becomes broader and more asymmetrical in shape. Its deconvolution becomes more complex, and for few-layer graphene (less than 10 layers), it may be fitted with three Lorentzian functions with a FWHM higher than 24 cm^−1^ each. For more than 10 layers, the 2D band becomes similar to that of graphite, and may be fitted with two Lorentzian functions [36,38]. Figure 5 shows the Raman spectra of the graphite starting materials and corresponding exfoliated graphite.

**Figure 6:**
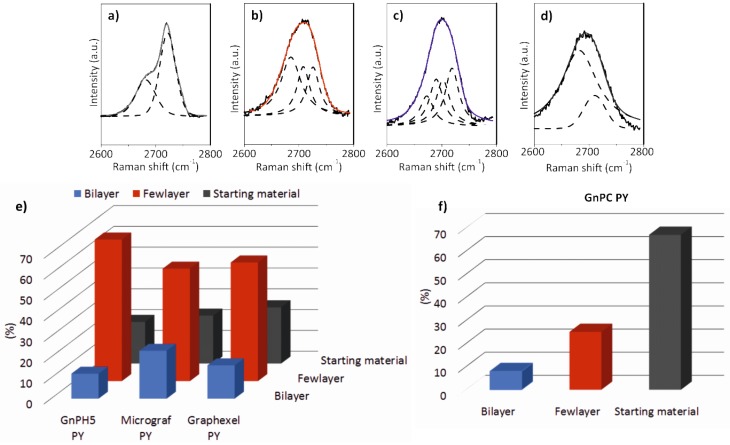
Lorentzian features of the deconvoluted 2D band of (**a**) graphite starting material (Graphexel), (**b**) few-layer graphene, (**c**) bilayer graphene, and (**d**) GnPC starting material; (**e**) yield of exfoliation process for Graphexel, Micrograf, GnPH5, and (**f**) GnPC.

The Raman spectra of the Graphexel, Micrograf, and GnPH5 starting materials (Figure 5a–c respectively) are typical of graphitic materials. The D band at 1350 cm^−1^ is almost absent compared to the G band (at 1581 cm^−1^), which indicates the good structural quality of the graphite. The 2D band position of Graphexel and GnPH5 occurs at approximately 2722 cm^−1^, and its shape is asymmetric, which is typical of the graphite 2D band [36]. The 2D band of Micrograf is less asymmetric and downshifted (~2718 cm^−1^), which is typical of a more exfoliated material. The 2D band of all of these starting materials can be fitted with two Lorentzian functions, as shown in Figure 5a. After the exfoliation process with PY in solution (Graphexel PY, Micrograf PY, and GnPH5 PY in Figure 5a–c) the Raman spectra show the presence of FLG and more highly exfoliated products such as bilayer graphene. The 2D band of FLG was identified by its deconvolution into three Lorentzian functions (Figure 6b). Bilayer graphene has a symmetric 2D band centered at 2702 cm^−1^, which was deconvoluted with four Lorentzian functions (Figure 6c). The D band of the exfoliated material shows a higher intensity than the corresponding graphite flakes, which may be due to the larger fraction of edge carbon relative to basal-plane carbon [23]. The characteristic PY peaks are also observed in the spectra.

Figure 5 shows the Lorentzian features of the deconvoluted 2D band of the graphite starting material, few-layer graphene and bilayer graphene, as well as the yield of the exfoliation process for Graphexel, Micrograf, GnPH5, and GnPC.

The Raman spectra of the GnPC starting material that was exfoliated with PY are presented in Figure 5d. The spectra show a prominent D band near 1350 cm^−1^, with slightly higher intensity than the G band (at 1581 cm^−1^), which may be related to the smaller flake size. Thus, it also has a higher edge-to-basal plane ratio, as well as a higher oxidation level, which is in agreement with the producer specifications and the TGA analysis. The 2D band position occurs near 2692 cm^−1^, showing high symmetry, although with lower intensity compared to the G band. The 2D band of the GnPC starting material can be fitted with two Lorentzian functions, as shown in Figure 5d. After the exfoliation process, a decrease of the D band intensity is observed, which may be due to the higher adsorption of PY on the less “defective” GnPC flakes (less oxidized), thus selectively stabilizing them in aqueous suspension.

A statistical analysis of the flakes formed in suspension by exfoliation of graphite in water using PY was performed by Raman spectroscopy. The analysis was based on the Raman spectra collected for 60 different flakes of each exfoliated material (Graphexel, Micrograf, GnPH5, and GnPC), and the spectra of bilayer and FLG were identified. Figure 5e shows the yield of bilayer, FLG, and remaining graphite starting material identified after the exfoliation process of Graphexel, Micrograf, and GnPH5. GnPC has a different Raman spectrum compared to the other graphite materials, with similarities with the spectrum of reduced graphene oxide [39]. The differences observed after PY exfoliation are less pronounced than those described for the other materials. The statistical analysis of the Raman 2D bands of the exfoliated GnPC (Figure 6f) show a large fraction of flakes with similar morphology as the starting material, but it also presents flakes that are typical of FLG and bilayer graphene [38].

The FLG and bilayer graphene spectra were identified based on the position of the 2D band and its deconvolution, and are illustrated in Figure 6b,c, respectively. The analysis demonstrated the extent of exfoliation into the bilayer graphene of 16%, 23%, and 12% for Graphexel, Micrograf, and GnPH5, respectively. FLG was identified in 57%, 54%, and 68% of exfoliated Graphexel, Micrograf, and GnPH5, respectively. Finally, the spectra that were identified as similar to the starting materials corresponded to 27%, 23%, and 20% of the analyzed materials from Graphexel, Micrograf, and GnPH5, respectively. The results are summarized in Figure 6e. Figure 6f shows the yield of bilayer, FLG, and starting material obtained after the exfoliation of GnPC. The statistical analysis of the 2D band of the GnPC PY spectra showed the formation of 8% bilayer, 25% FLG, and 67% of starting material.

It should be pointed out that the GnPC starting material is itself a highly exfoliated material, as its 2D spectrum is quite different from that of graphite (Figure 6d). Jang et al. [40] reported a similar Raman spectroscopic analysis of 105 different sample spots to identify monolayer graphene and FLG obtained from graphite, which was exfoliated using 1-pyrene sulfonic acid sodium salt and supercritical fluid ethanol. Schlierf et al. [22] also reported a similar study for the exfoliation of graphite in water using different pyrene derivatives. The Raman spectroscopic analysis was performed on 60 to 70 different flakes. The authors obtained an exfoliation yield of 86% (a total of single-layer to few-layer) using a higher concentration of pyrene derivative (0.33 mM) and longer sonication time (5 h to 35 h) compared to the conditions used in the present work. 

**Figure 7:**
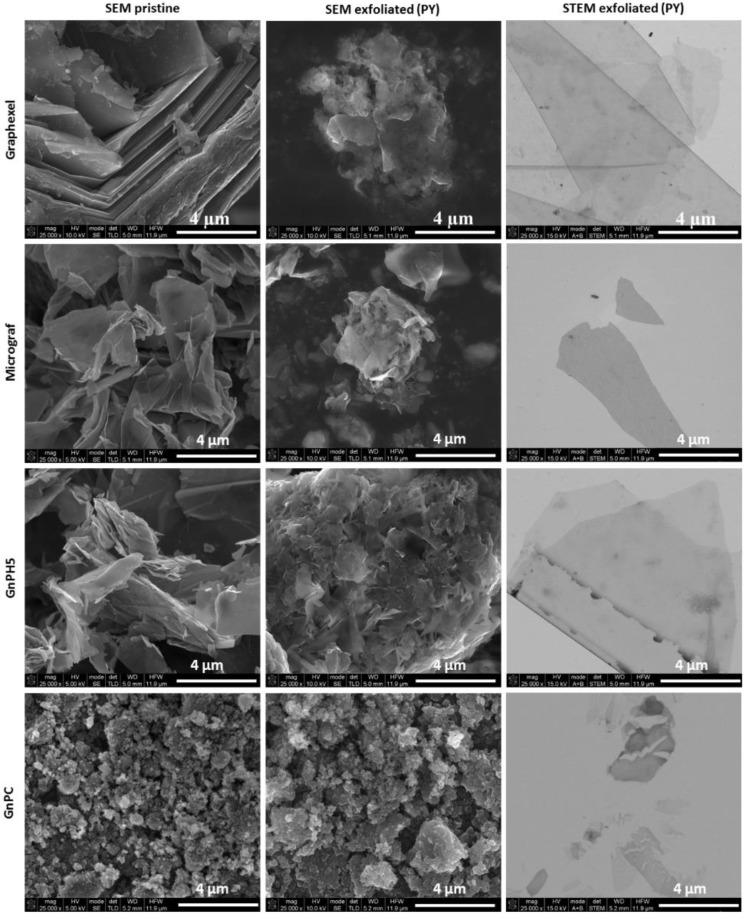
Scanning electron microscopy (SEM) and scanning transmission electron microscopy (STEM) of the graphite starting material and exfoliated graphite.

The morphology of the graphite materials and corresponding PY exfoliated materials is illustrated in Figure 7, showing the SEM of the powders and STEM of samples collected from the corresponding aqueous suspensions. The SEM images for the exfoliated Graphexel, Micrograf, and GnPH5 show apparently thinner flakes compared to the starting material, while GnPC shows a similar morphology before and after exfoliation. The STEM images of all of the exfoliated graphite grades show the formation of thin flakes.

### 3.4. Study of the Pyrene Derivative/Exfoliated Graphite Interactions

The Raman spectroscopy studies reported above demonstrated that the application of energy to graphite in a PY aqueous solution produces a GnP stable suspension with more that 70% of the suspended GnP flakes exfoliated into FLG. The question arises now concerning the nature of the interactions between PY and the FLG surface. In particular, how PY is adsorbed at the FLG surface, how many PY molecules can be accommodated on a given area, and if they form a monolayer or stacks of several adlayers? Also, does PY tend to stack and form organized structures itself? In order to answer these questions, we used X-ray diffraction (XRD) and molecular modeling studies. X-ray diffraction studies were performed on a set of materials produced from FLG/PY suspensions by water evaporation; molecular modeling studies were performed that helped understanding the nature of the graphene/PY interactions.

#### 3.4.1. X-ray Diffraction

**Figure 8:**
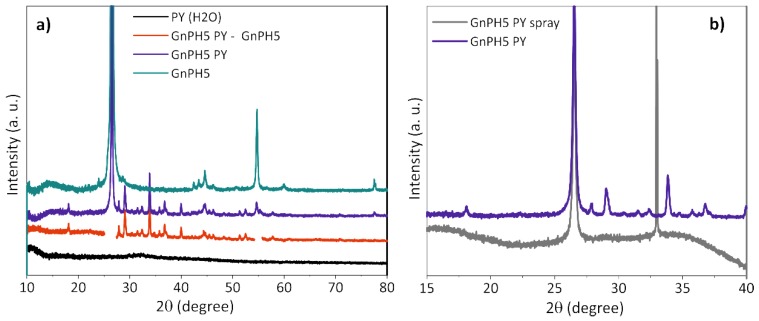
X-Ray intensity profiles of GnPH5 and PY, as well as the exfoliated GnPH5 PY (**a**), as a powder formed by solvent evaporation from the bulk suspension or by spray deposition on a Si wafer (**b**).

A GnPH5 PY suspension was prepared as described in the experimental section, and the GnPH5 PY powders were obtained by water evaporation from the bulk suspension in an oven at 90 °C. A PY solution with the same concentration used for exfoliation was prepared, and the PY powder was collected by solvent evaporation. The XRD profiles presented in Figure 8a were acquired for as received GnPH5, GnPH5 exfoliated in PY solution, and PY, all in powder form, and the last two obtained after solvent drying. The GnPH5 shows two dominant peaks at 26.57° 2θ and 54.74° 2θ, and lower intensity peaks at 42.5° 2θ, 43.4° 2θ, 44.5° 2θ, and 77.5° 2θ, which was similar to a graphite structure. The GnPH5 PY had peaks at the same positions as observed for GnPH5, and a significant number of additional diffraction peaks. The subtraction of the GnPH5 XRD profile from the GnPH5 PY is also shown in Figure 8a for a clearer view of the additional peaks observed. These peaks originate on structures that were formed upon slow-drying of the dispersed GnPH5 PY.

The PY in powder form, which was obtained after dissolution in water and drying, does not display any peaks in the XRD difractogram, showing that this pyrene derivative does not form organized structures upon solvent evaporation. Thus, the results obtained indicate that when FLG and PY molecules are present in solution, an ordered structure is formed additionally to that observed for the original GnPH5. If these structures originate on PY organization/stacking, then they must be formed from several PY adlayers in order to produce a 3D crystal, and grow from the FLG surface.

The same GnPH5 PY suspension was used to prepare a sample by spray drying that was also analyzed by XRD. Figure 8b compares the X-ray profile of the GnPH5 PY powder obtained by slow solvent drying from the bulk suspension with that obtained by spraying the same suspension onto a heated Si wafer. The latter depicts only one intense diffraction peak at 26.52° 2θ, suggesting the presence of the graphitic ordered structure from the GnPH5 with an interlayer spacing of 0.336 nm. The intense peak at around 33.5° 2θ is due to the Si wafer substrate.

The deposition/solvent drying method may strongly influence the molecular organization in the powder formed. Here, the additional XRD peaks observed on GnPH5 PY from slow drying, which are absent on the GnPH5 PY obtained by fast drying, indicate that PY stacking occurred from solution only under slow-drying conditions.

#### 3.4.2. Molecular Modeling

A graphene hexagonal flake possessing a closed-shell electronic ground state, 546 carbon atoms, and 5-nm radii was chosen as the substrate. The use of flakes, as opposed to periodic systems, allows the explicit study of edge effects, the possibility of side switching, and the possibility of solvated systems. The adsorption of a variable number of the functionalized pyrene molecules, namely 1, 2, 4, 6, 7, 8, 9, 10, 11, and 12, was computed. For this, we performed molecular dynamics (MD) simulations using the molecular mechanics MMFF94 force field as implemented in the software Tinker. For simplicity and computational efficiency, the graphene substrate was held fixed during the classical simulations. A simulated annealing technique with initial and final temperatures of 500 K and 300 K respectively was chosen in order to improve the sampling of the potential energy surface during the simulations. Simulations were run for 20 ns with a RESPA integrator that allowed the use of a time step of 2 fs. Snapshots of the trajectory were selected at 2 ps intervals, yielding 10,000 different adsorption geometries that were optimized with the MMFF94 force field. Two representative geometries for each system were selected; geometries with the lowest total energy and geometries with the lowest intermolecular energy and optimized at the PM6-D3H4 level with the program MOPAC16 in vacuum, giving comparable results [41]. 

The relative position of the adsorbate with respect to the graphene flake was monitored through the MD simulations by following the Cartesian coordinates of the nitrogen atom of PY, as nitrogen atoms with positions up to 5 Å from the flake plane were found to correspond to PY molecules directly adsorbed onto the surface. The typical number of adsorbed neutral pyrene molecules during the MD simulations was found to be eight. The maximum number of molecules that this flake can accommodate in one adlayer was found to be 10, but this was seldom found during the MD simulations. The geometries for the lowest energy minima found at the MMFF94 level were optimized at the PM6-D3H4 level (not shown) and at the GFN-xTB level methods for PY in its neutral (carboxylic) and anionic deprotonated (carboxylate) states; these are presented in Table 2. The GFN-xTB (Geometry, Frequency, Non-covalent, eXtended Tight-Binding) method is a recent semiempirical method developed by Grimme et al. that allows systems with thousands of atoms within a water continuum model to be computed efficiently [42].

For neutral PY in vacuum, the binding energy changes considerably, from −38 kcal/mol for 1 adsorbed PY to ~50 kcal/mol for 4 PY and 8 PY adsorbed molecules, respectively. This is due to the attractive interaction between PY molecules, which peaks for 4–8 PY molecules. When the carboxylate form of PY is considered, the binding energy for one molecule, −39 kcal/mol, is similar to the binding energy for neutral PY. However, in this case, it does not change with the number of adsorbed molecules, as in negatively charged PY adsorbates and the electrostatic repulsion counterbalances the hydrogen bonding and dispersion attractions. The differences between the neutral and the anionic form of PY are clear when the graphene flake is saturated with 10 PY molecules. In the neutral form, the lowest energy structure accommodates 10 PY molecules in more than one adlayer, while in the anionic form, the opposite is observed, i.e., one adlayer is more stable, as electrostatic repulsion inhibits the deposition of more PY adlayers.

**Figure 9:**
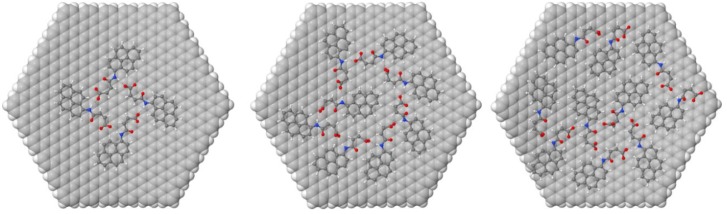
Models with four, eight, and 10 deprotonated PY molecules adsorbed on a graphene monolayer flake at the GFN-xTB level. The graphene flake is represented by an array of carbon spheres, and the PY molecules are shown with the ball and stick representation.

The optimized geometries at the GFN-xTB level for four, eight, and 10 deprotonated PY molecules in water are presented in Figure 9. It should be noted that a symmetrical structure is found only for four PY molecules, i.e., at low coverage, and that there is not a clear adsorption pattern for eight PY and 10 PY molecules. In addition, the largest concentration in the simulations where there is hydrogen bonding connecting all of the adsorbed molecules is for eight PY molecules per graphene flake. Both observations suggest that long-range ordering might be frustrated for PY molecules on graphene.

**Figure 10:**
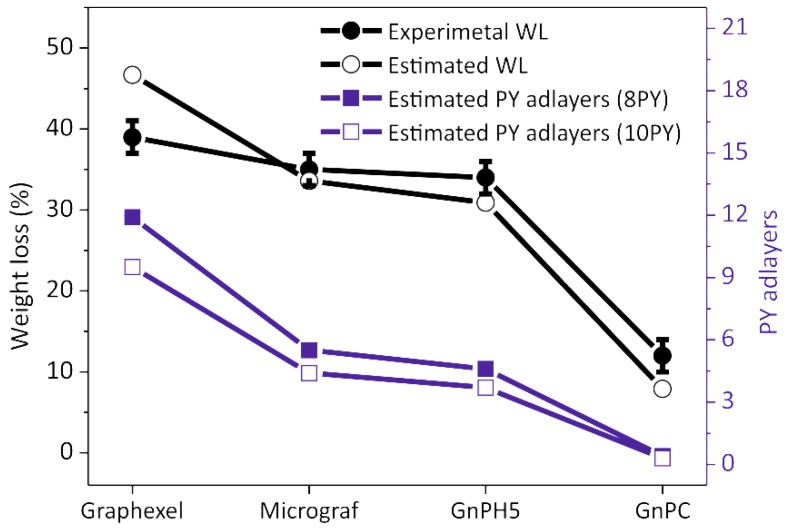
Experimental and estimated TGA weight loss for the exfoliated materials and estimated number of PY adlayers.

The experimental TGA weight loss (WL) results have been compared with the estimate calculated considering the mass of PY in solution and the exfoliation yield of carbon in suspension (from UV-visible spectroscopy); these are presented in Figure 10. The number of PY adlayers that can be accommodated on the FLG surface during the slow drying of the solution under the conditions mentioned, was estimated for the exfoliated materials. The average number of graphene layers in the FLG, as estimated by Raman statistical analysis (Figure 5), and the amount of carbon in suspension, as measured by UV-vis, were used to estimate the total surface available for adsorption. The estimate was carried out considering two of the model conditions described in Figure 9, namely that the graphene flake defined in the computational model carries eight (8PY) or ten (10PY) PY molecules on each flake side.

The calculations showed that the experimental WL is close to the estimated WL for all of the materials, considering that all of the pyrene derivative remains in solution. During solvent evaporation, the PY molecules may deposit randomly or in a regular manner, with the latter leading to the formation of stacked PY adlayers at the surface of the exfoliated material [24,25]. These organized adlayers are likely to be the origin of the XRD diffraction peaks observed in the FLG-PY obtained from slow solvent evaporation. Considering that the maximum number of PY molecules that a model graphene can accommodate is eight or ten, the number of PY adlayers was estimated for the FLG formed from Graphexel, Micrograf, GnPH5, and GnPC resulting in, respectively, 11.9, 5.5, 4.6, and 0.4 for 8PY, and 9.5, 4.4, 3.7, and 0.3 for 10PY (Figure 10). That the estimated number of PY adlayers for the GnPC FLG is below 1 reflects the large degree of exfoliation attained for this material, and thus the large available flake surface for a small PY concentration in solution.

## 4. Conclusions

A pyrene derivative was synthetized through a simple and low-cost functionalization methodology that leads to a high yield of the final compound, and can potentially be scaled up for large-scale production. The solubility of this pyrene derivative in water allowed its use for the exfoliation and stabilization of different types of graphite in aqueous media. The statistical Raman spectroscopic analysis showed the formation of bilayer and few-layer graphene after the exfoliation process of all of the types of graphite, and the STEM images showed the formation of thin flakes, in agreement with Raman spectroscopy. The XRD results showed the d-spacing of the larger FLG materials, similar to the graphite, and the molecular modeling demonstrated the maximum number of PY molecules that can be adsorbed to the surface of graphene. The estimated weight loss in the TGA analysis is similar to the experimental values, suggesting an excess of PY molecules adsorbed on the exfoliated material in more than one adlayer. 

The very low concentration of pyrene derivatives used in this work (lower than 16 ppm, to our knowledge, the lowest reported in the literature) to exfoliate graphite in water opens up the possibility of an efficient and cost-effective process to produce FLG. The application in biomedical areas requires further studies on the toxicity and biocompatibility of this new pyrene derivative. The FLG may be deposited directly from the aqueous suspension using techniques such as spray or spin-coating, or in the form of composites with water-soluble polymers or stable aqueous suspensions of polymers. The FLG may be collected from the aqueous suspensions by solvent evaporation and used in powder form in the production of polymer composites by melt-mixing methods.

## Figures and Tables

**Table 1:** Yield of graphite exfoliation reported in the literature and the results obtained in the present work.

Starting Material	Equivalent Diameter (µm)	Pyrene Derivative Concentration (mM)	Sonication Time (h)	Yield of Exfoliation (%)	Reference
Graphite + 1-pyrenecarboxylic acid	45	1.3	24.0 **^a^**	1.0	[17]
Expanded graphite + Sodium 1-pyrenesulfonate	15	3.0	1.0 **^b^**	2.0	[16]
Natural Graphite + Sulfonate substituted pyrene	45	6.0	1.0 **^b^**	2.4	[20]
Graphite + 1-pyrenecarboxylic acid	50	10.0	1.5 **^a^**	2.5	[19]
Graphite (Graphexel)	180	0.05	1.0 **^b^**	2.0	Present work
Micronized graphite (Micrograf)	10	0.05	1.0 **^b^**	4.0	
Expanded graphite (GnPH5)	5	0.05	1.0 **^b^**	5.0	
Expanded graphite (GnPC)	1–2	0.05	1.0 **^b^**	85	

**^a^** bath sonication. **^b^** tip sonication.

**Table 2:** Computed binding energies per deprotonated and neutral PY molecules adsorbed on the graphene flake and corresponding number of C atoms per molecule. All of the energies are in kcal/mol.

	E_Binding_	
# PY	PY (Carboxylate) in Water	PY (carboxylic) in Vacuum
**1 (1 adlayer)**	−39	−38
**4 (1 adlayer)**	−39	−50
**8 (1 adlayer)**	−39	−49
**10 (1 adlayer)**	−38	−46
**10 (>1 adlayer)**	−35	−48
**12 (>1 adlayer)**	−36	−47

## Supplementary Materials

The following are available online at http://www.mdpi.com/2079-4991/8/9/675/s1.
