# Supplementary material for: Water Dispersible Few-Layer Graphene Stabilized by a Novel Pyrene Derivative at Micromolar Concentration
Source: Nanomaterials (Basel). 2018 Aug 30;8(9):675. doi: 10.3390/nano8090675 (PMC6163987; doi:10.3390/nano8090675)
Supplement: Supplementary file 1 [file nanomaterials-08-00675-s001.pdf]

Supporting information

Synthesis of the pyrene derivative

The NMR spectra were recorded on a Varian Unity Plus ($_1$H: 300MHz, ^{13}C: 75MHz) or on a Bruker Advance III 400 (^{1}H: 400Mz, ^{13}C 100 Mz) including the ^{1}H – ^{13}C correlation spectra (HSQC). Deuterated DMSO-d_6 was used as a solvent. The chemical shifts are expressed in δ (ppm) and the coupling constants, *J*, are reported in hertz (Hz). The peak patterns are indicated as follows: s, singlet; d, doublet; t, triplet; m, multiplet; q, quartet and br, broad. Fourier transform infrared spectra were recorded on a Spectrum 100 Perkin-Elemer apparatus in transmission mode using NaCl pellets.

1-Nitropyrene (1) was prepared according to a reported procedure. [29] Briefly, to a mixture of pyrene (4.0 g, 19.8 mmol) and Ac_2O (5.2 mL, 55.4 mmol) in EtOAc (50 mL) was added $Cu(NO_3)_2.3H_2O$, (6.2 g, 27.7 mmol). The mixture was stirred at 55ºC for 20 h, and a thick yellow precipitate formed. The reaction mixture was cooled to room temperature and the inorganic materials were filtered off. The crude product obtained on evaporation of the solvent was purified on a silica gel column (3.2 cm x 19 cm, 10-60% CH_2Cl_2/ petroleum ether) to give the pure product (3.68 g; 75%). The FTIR spectrum is presented in **Figure S1.**

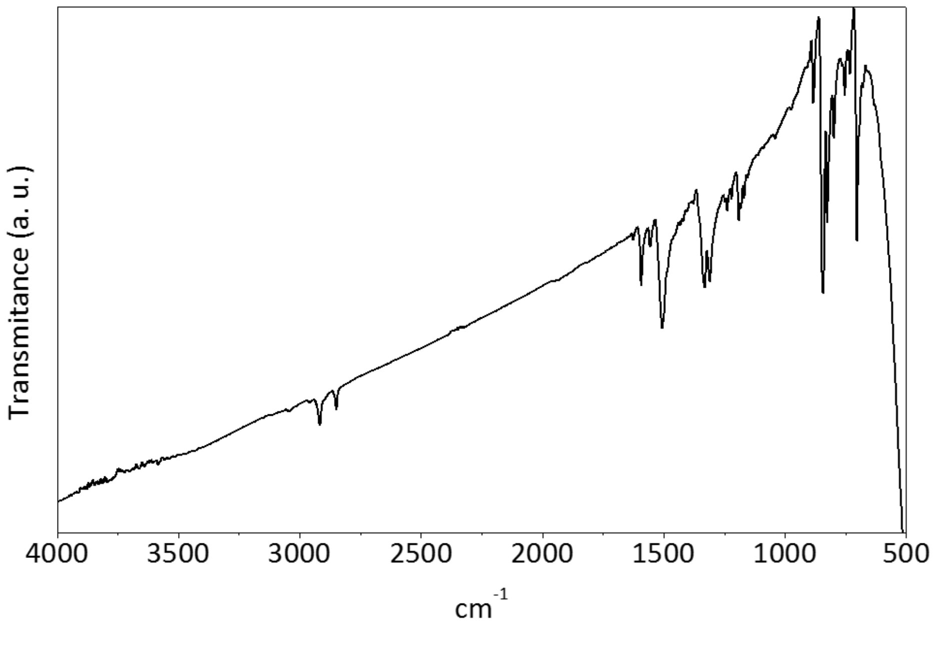

S1: FTIR spectrum of the 1-Nitropyrene (1)

1-Aminopyrene (2): 1-Nitropyrene (1) (13.5 mmol,3.3 g) was dissolved in ethanol (200 mL), the mixture cooled in an ice bath, and copper (II) sulfate (32.4 mmol, 5.2 g) was added. After stirring for 10 min, sodium borohydride (67.5 mmol, 3.8 g) was added slowly during 15 min. The mixture was stirred under reflux overnight. After being cooled to room temperature, the solvent was removed. The reaction mixture was

dissolved in dichloromethane (100 mL), and water (100 mL) was added. The aqueous layer was extracted with dichloromethane (3 x 30mL), and the organic layers were washed with brine (2 x 30 mL) and dried with $MgSO_4$. The removal of the solvent in vacuum gave compound **2** (2.41 g, 82%). ^{1}H NMR (400 MHz, DMSO-d_6): δ (ppm) 6.32 (s, 2H, -NH2), 7.40 (d, J = 8.3 Hz, 1H, H2), 7.71 (d, J = 8.8 Hz, 1H,H5), 7.86 (t, J = 7.7 Hz, 1H, H7), 7.88 (d, J = 8.8 Hz, 1H, H4), 7.92 (d, J = 9.3 Hz, 1H, H10), 7.97 (d, J = 8.4 Hz, 1H, H2), 7.98 (d, 1H, H6), 7.99 (d, 1H, H8), 8.29 (d, J = 9.2 Hz, 1H, H9). ^{13}C RMN (100 Mz, DMSO-d6): δ (ppm) 113.13 (C2), 114.72 (C3^{a}), 121.39 (C10^{a}), 121.76 (C5), 122.13 (C9), 122.37 (C6), 122.91 (C8), 124.14 (C10), 125.14 (C3^{a1}), 125.70 (C5^{a1}), 125.85 (C7), 126.52 (C3), 127.70 (C4), 131.67 (C8^{a}), 132.02 (C5^{a}), 144.36 (C1). The RMN spectra are presented in **Figure S2**.

a)

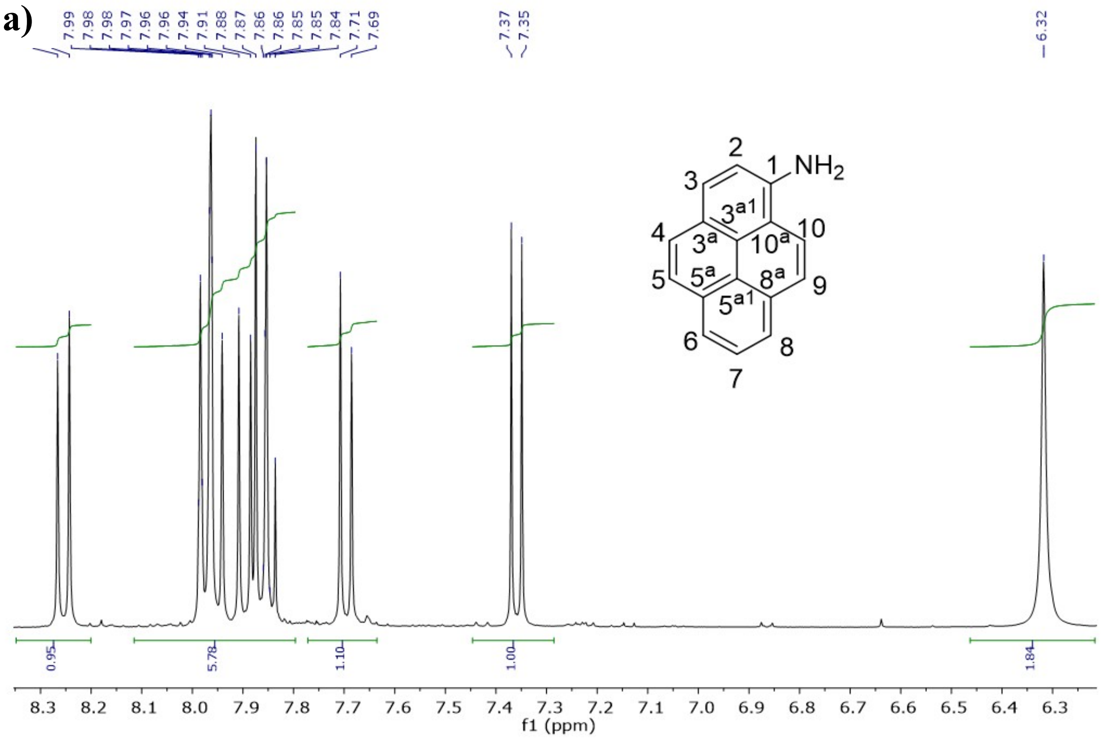

b)

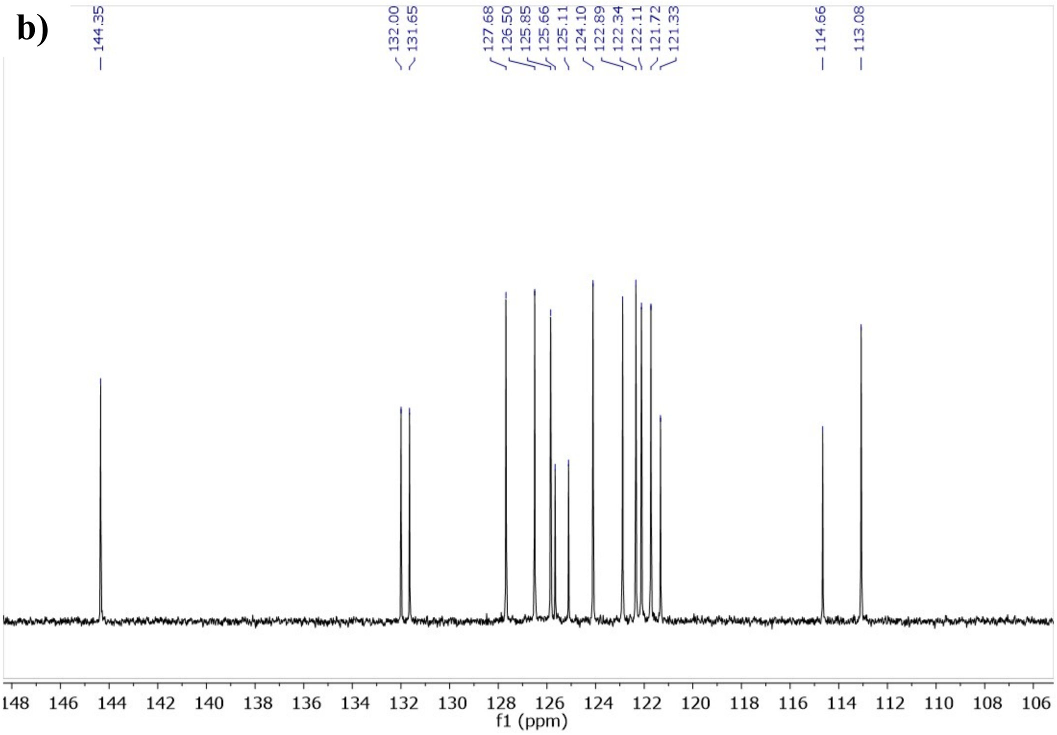

c)

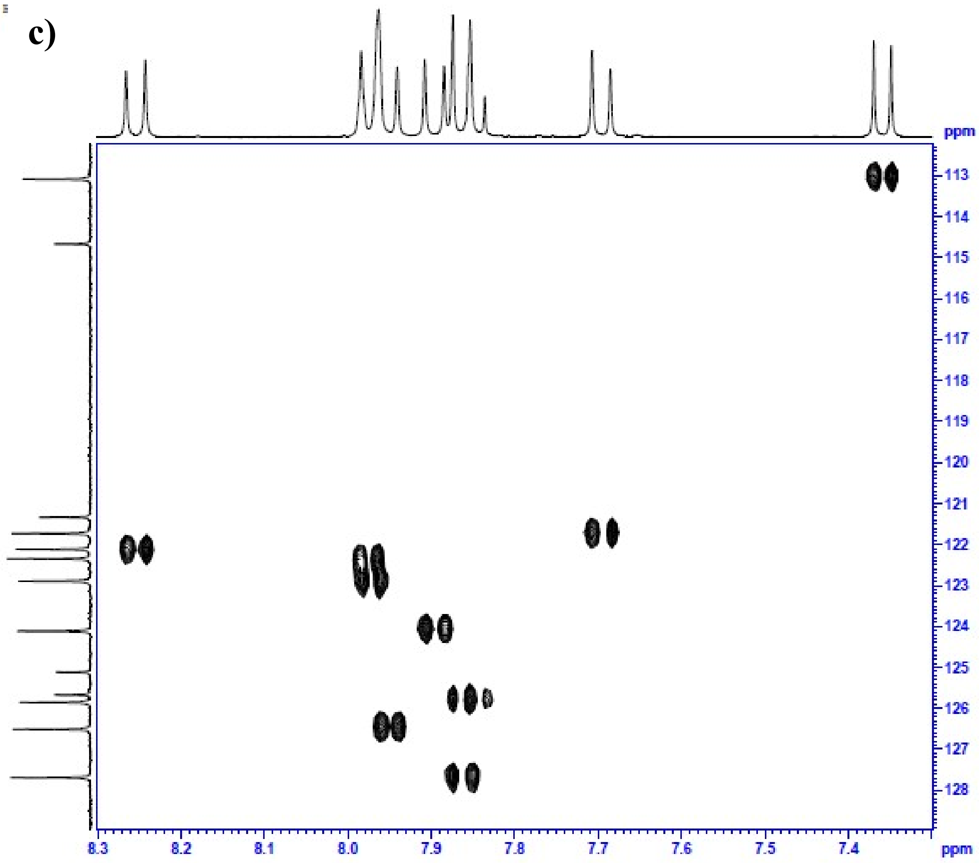

S2: RMN spectra of 1-Aminopyrene (2): a) ^{1}HRMN; b) ^{13}C NMR; and c) 2D HSQC (heteronuclear single quantum correlation).

The FTIR spectrum is presented in **Figure S3**.

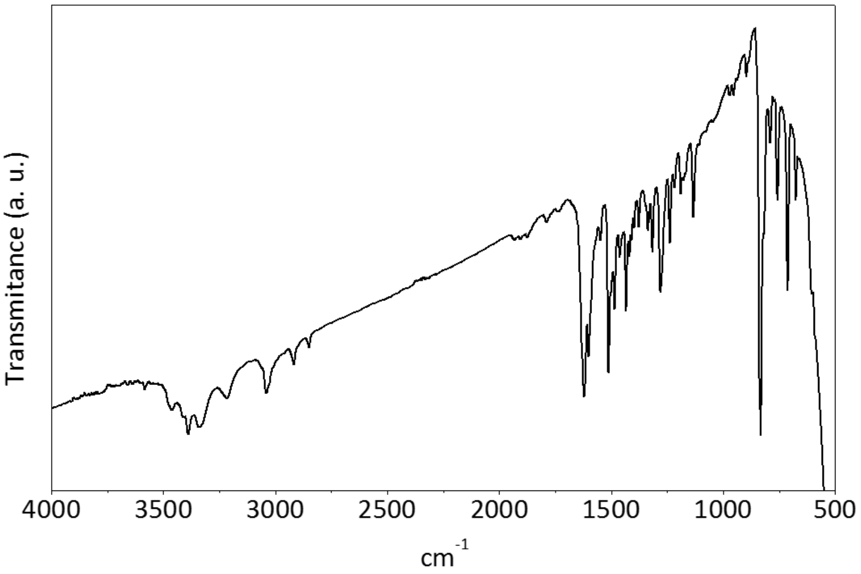

S3: FTIR spectrum of the 1-Aminopyrene (2)

(Z)-4-oxo-4-(pyren-1-ylamino)but-2-enoic acid (3): To a solution of 1-aminopyrene (11.1 mmol, 2.4 g) in acetonitrile (200 mL), maleic anhydride (16.6 mmol, 1.6 g) was added and the mixture was stirred at room temperature. The reaction followed by TLC (20% ethyl acetate/petroleum ether) and after four hours, the precipitate was filtered. Compound **3** (3.0 g, 86%) was obtained as a yellow solid. ^{1}H NMR (400MHz, DMSO-d_6): δ (ppm) 6.43 (d, 1 H, *J* = 12.4 Hz, CH), 6.77 (d, 1 H, *J* = 12.4 Hz, CH), 8.08 (t, 1 H, *J* = 7.6 Hz, H7), 8.13-8.18 (m, 2 H, H5 and H10), 8.22 (d, 1 H, *J* = 9.2 Hz, H4), 8.29-8.34 (m, 4 H, H2, H3, H6 and H8), 8.41 (d, 1 H, *J* = 9.2 Hz, H9), 10.84 (s, 1 H, NH), 13.27 (s broad, 1 H COOH). ^{13}C RMN (100 Mz, DMSO-d6): δ (ppm) 122.45 (C9), 123.19 (C2), 123.84 (C3a), 124.04 (C3a1), 124.36 (C5a1), 124.98 (C3), 125.05 (C6 or C8), 125.34 (C6 or C8), 126.47 (C7), 126.79 (C5 and C10), 128.54 (C10a), 130.49 (C8a), 130.83 (C5a), 130.85 (CH), 131.10 (C1), 164.53 (COOH), 167.05 (C=O).
The RMN spectra are presented in **Figure S4.**

a)

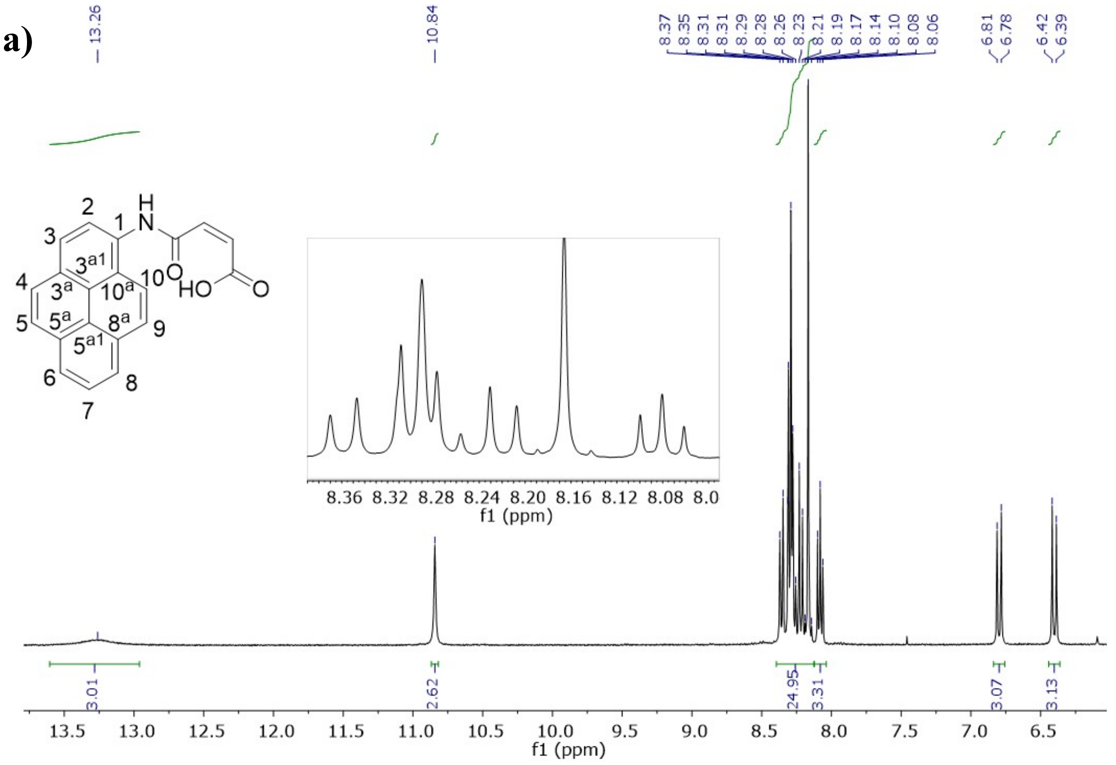
13.26
10.84
8.37 8.35 8.31 8.31 8.29 8.28 8.26 8.23 8.21 8.19 8.17 8.14 8.10 8.08 8.06
6.81 6.78
6.42 6.39
3.01
2.62
24.95
3.31
3.07
3.13
f1 (ppm)

b)

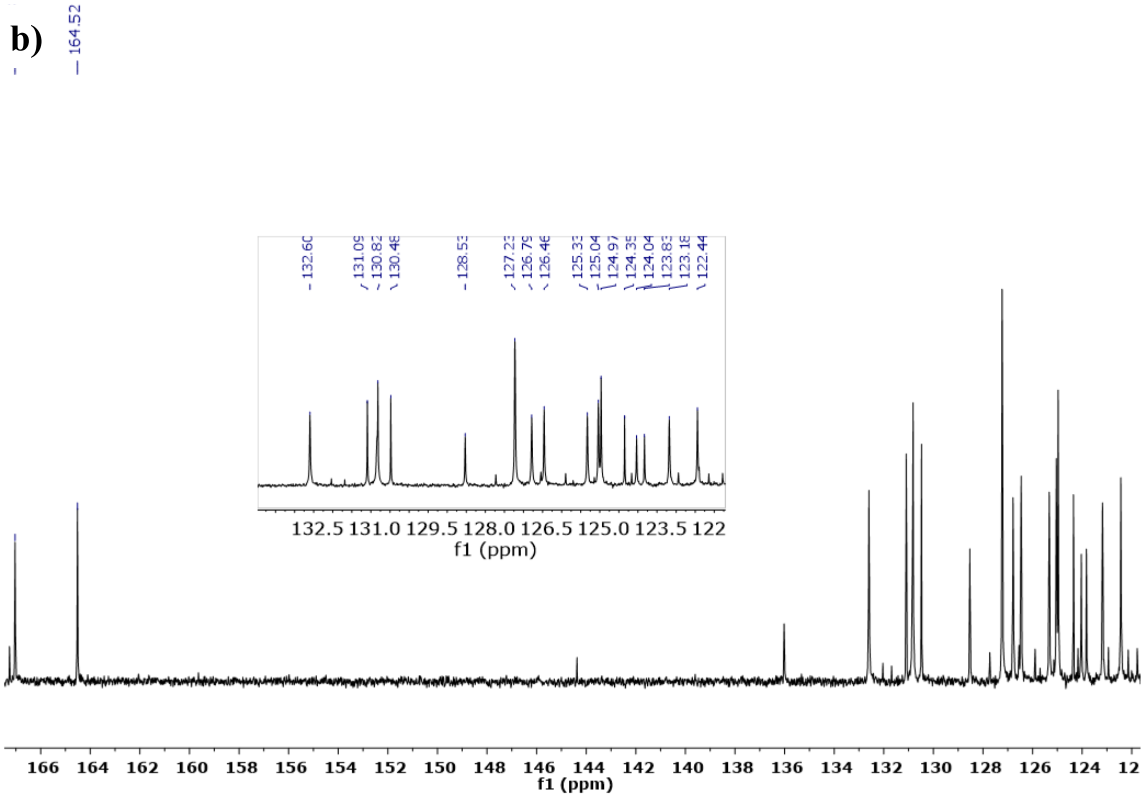
164.52
132.60 131.09 130.82 130.48 128.53 127.23 126.79 126.46 125.33 125.04 124.97 124.39 124.04 123.83 123.18 122.44
f1 (ppm)

c)

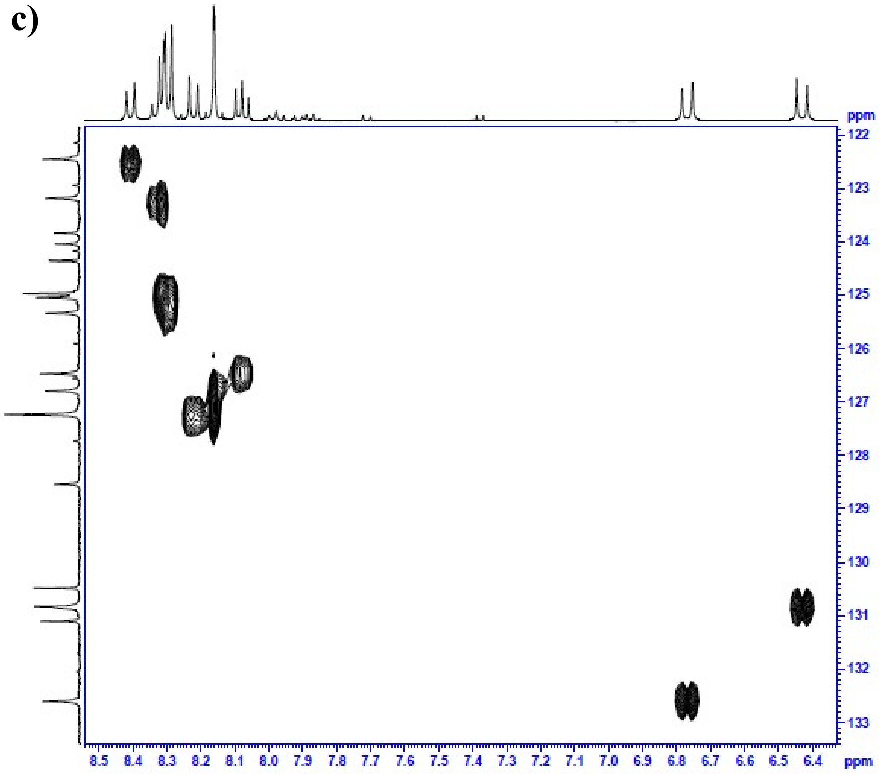

S4: RMN spectra of (Z)-4-oxo-4-(pyren-1-ylamino)but-2-enoic acid (3): a) ^{1}HRMN; b) ^{13}C NMR; and c) 2D HSQC (Heteronuclear Single Quantum Correlation).

The FTIR spectrum is presented in **Figure S5.**

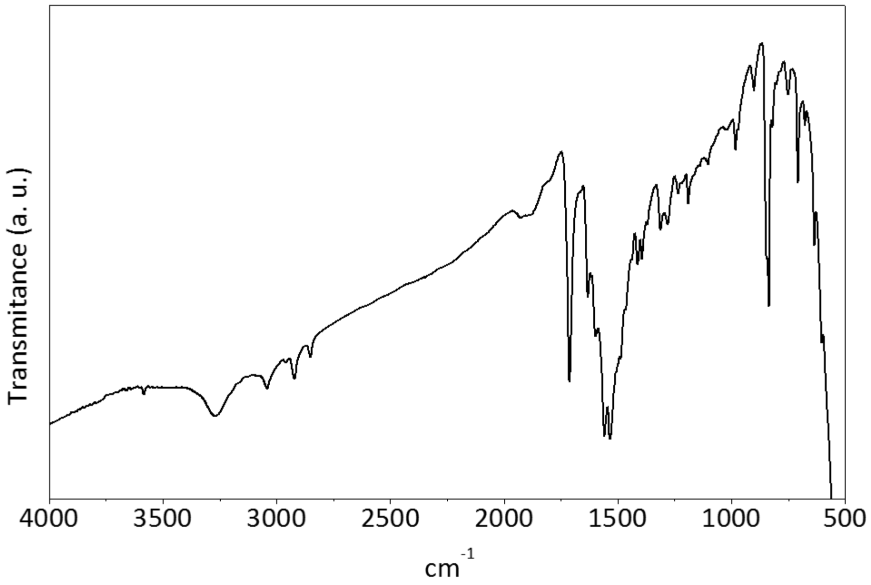

S5: Fourier transform infrared (FTIR) spectrum of the (Z)-4-oxo-4-(pyren-1-ylamino) but-2-enoic acid (3)
